# Using Human-Centered Design Strategies to Identify Unmet Adolescent Sexual Health Wants and Needs

**DOI:** 10.1007/s11121-023-01559-5

**Published:** 2023-06-23

**Authors:** Cristina Leos, Elizabeth Chen, Vichi Jagannathan

**Affiliations:** 1 MyHealthEd, Inc, Chapel Hill, USA; 2 Department of Health Behavior, Gillings School of Global Public Health, The University of North Carolina at Chapel Hill, Chapel Hill, USA; 3 Rural Opportunity Institute, Rocky Mount, USA

**Keywords:** Human-centered design, Intervention development, mHealth, Adolescent health, Sexual health

## Abstract

This paper describes a human-centered design approach to investigate unmet adolescent sexual health wants and needs among youth of color in low-income and rural communities in Texas, North Carolina, and Connecticut (*n* = 149). The approach involved gathering qualitative data through in-depth interviews and other human-centered design activities between April 2016 and August 2016. Data were analyzed using thematic content analysis after each round of data collection. Themes were triangulated across multiple data sources to identify key insights. Results highlighted several important unmet wants and needs. Participants shared that schools were not the preferred place to learn about sexual health and healthy relationships, due to uncomfortable and sometimes even hostile environments. When discussing the potential to use internet- and technology-based sexual health resources, participants expressed concerns over privacy, safety, and credibility of information available. Similarly, participants preferred mobile apps over websites due to the privacy of the experience. Most importantly, key emotions impacting adolescents’ access to and use of sexual health resources were identified. Results indicated a preference for consuming story-based information in a text message format that described diverse experiences related to sexual health topics. Together, these findings led to the development of an innovative, mobile health intervention for adolescents, the *Real Talk* mobile app. This human-centered approach can support researchers and practitioners in strengthening intervention development efforts to improve the reach, adoption, and implementation of sexual health interventions.

**Supplementary Information:**

The online version contains supplementary material available at 10.1007/s11121-023-01559-5.

## Introduction

Millions of school-aged adolescents in the USA do not receive sex education in schools (National Conference of State Legislatures, 2020), despite evidence indicating its effectiveness in improving sexual health outcomes (e.g., McKay et al., 2021). This is due to multiple barriers to accessing and implementing high-quality sexual health programs in schools, including local, state, and national policy, limited curricular options, and lack of school resources and training (Eisenberg et al., 2012; Leos & Wiley, 2020). Notably, these barriers have produced significant and persistent disparities in receipt of comprehensive sex education for Black, Latinx, and Indigenous youth as well as those living in rural areas (Lindberg et al., 2016; Phillips et al., 2020), communities which already face disproportionate rates of sexually transmitted infections and teen births. These disparities demonstrate the need for continued efforts to expand access to high quality sex education for Black, Latinx, Indigenous, and rural youth in the USA.

Digital health interventions have emerged as one strategy for overcoming key barriers to accessing sexual health information. These programs can be implemented and disseminated at lower cost and offer more relevant and personalized content compared to in-person interventions (Kreps & Neuhauser, 2010). Existing digital sexual health interventions have demonstrated effectiveness at increasing knowledge, safer sex norms, attitudes, and condom use (Widman et al., 2018). Technology-based interventions may also help address disparities considering the widespread access to smartphones and internet usage, even among rural, low-income, and Black and Latinx communities (Vogels et al., 2022). This positions digital interventions as effective strategies to offset otherwise unequal access to sex education in schools and other settings. However, despite the promises of digital interventions, adolescents themselves are often not involved in the development process (Gavine et al., 2016). This results in programming that does not meet their wants and needs, reducing the potential reach, uptake, and overall impact of these efforts (Bailey et al., 2015). This represents an urgent gap in the sexual and reproductive health (SRH) field that must be addressed to effectively reduce sexual health disparities.

Human-centered design (HCD) may help fill this gap. Human-centered design is a creative problem-solving approach that begins by engaging deeply with the intended beneficiaries of a product or service to understand core wants and needs (IDEO.org, 2015). Human-centered design can be applied in combination with other approaches including community-based participatory research and implementation science (Chen et al., 2020, 2021). There are several HCD frameworks, one of the most well-known was developed by the global design firm IDEO (Brown & Wyatt, 2010). The HCD process begins by cultivating empathy for the intended beneficiaries of a product or service by exploring their challenges, desires, and opportunities for innovation. This includes real-world observations and immersive experiences to gain new insights on the problem (IDEO.org, 2015). Next, HCD calls for creative brainstorming and generating prototypes (low-cost, shareable versions) of early ideas to quickly gather feedback and refine potential solutions (IDEO.org, 2015). Prototyping is similar to pilot testing, with the important distinction that prototyping occurs with much greater frequency and over shorter cycles, resulting in more acceptable and effective solutions compared to non-iterative processes (Dow & Klemmer, 2011). This approach allows stakeholders to validate solutions throughout the process and uncover unknown implementation challenges that would threaten the acceptability and viability of the final solution. These activities continue to refine solutions until they are ready for wide-scale rollout and evaluation (IDEO.org, 2015).

In this study, HCD was applied to investigate unmet adolescent sexual health wants and needs among youth of color living in low-income and rural communities in Texas, North Carolina, and Connecticut. The goal of this project was to conduct formative research to develop a technological innovation to reduce disparities in access to high quality sex education. Through gathering qualitative data and testing innovative sex education concepts, the *Real Talk* mobile app was developed as a novel, mobile, sexual health intervention for adolescents.

## Methods

### Study Design

This is a qualitative, descriptive study performed for the purpose of intervention development. It was reviewed by the Institutional Review Board of the University of North Carolina at Chapel Hill and deemed exempt from human subjects research.

### Sample

The sample for this study included adolescents 11–18 years old from low-income and/or rural communities in Connecticut, North Carolina, and Texas. Different samples were recruited for each of the data collection methods described in the sections below.

From April to May 2016, purposive sampling was used to recruit adolescents (*n* = 9) as well as adults (e.g., teachers, coaches, parents, school administrators, *n* = 5) for one-on-one interviews. This sample was identified by applying the Extremes and Mainstreams recruitment method, which aims to learn from both typical (i.e., mainstream) and atypical (i.e., extreme) stakeholders for a particular design challenge (IDEO.org, 2015). Participants are identified based on what typical users say, think, or do, then identifying criteria that could be considered extreme. Participants for this study were selected based on (a) use of social media (individuals with frequent social media use and individuals with no social media use), (b) experience with romantic relationships (individuals with multiple reported romantic relationships and individuals with no prior romantic relationships), and (c) existing knowledge of or comfort with sexual health topics (individuals who self-reported a lot of knowledge/comfort and individuals who self-reported little knowledge/comfort). Participants were recruited with support from local community partners at each site. For example, we worked with school administrators, coaches, and teachers to identify participants and schedule interviews.

From June to August 2016, a convenience sample of youth of color from the same sites (*n* = 135) were recruited to participate in prototyping (i.e., concept testing) and co-creation activities through public libraries, youth centers, and schools with outreach support from local community partners at each site.

### Data Collection

This study used a variety of HCD methods including interviews with extremes and mainstreams, card sorts, analogous experiences, rapid prototyping, and co-creation sessions. These methods were conducted beginning with interviews with extremes and mainstreams, card sorts, and analogous experiences to learn from youth; their experiences related to sex education; and to generate insights. The next methods used were rapid prototyping and co-creation sessions to create and test concepts aligned with the insights generated. Some are commonly used methods (e.g., interviews), others are unique to HCD (e.g., analogous experiences, rapid prototyping) (IDEO.org, 2015). These methods are explained further in the following sections.

### Interviews with Extremes and Mainstreams

Qualitative interviews are an integral part of HCD (IDEO.org, 2015). The purpose of these interviews is to build empathy for individuals experiencing the challenge, by grounding in the words and stories that characterize their experiences. The HCD interview is designed to understand behaviors by focusing on storytelling, lived experience, specific examples, and emotions rather than hypothetical scenarios. Interviews also provide the opportunity to work through exercises (e.g., card sorts) to better understand sentiments individuals may struggle to articulate. The interview guide is available in Appendix A. Interviews were 45–60 min long and were conducted in-person in the participant’s home or in public settings (e.g., schools, libraries) by one of the co-authors. The co-authors conducting the interviews took field notes during the interview per the analysis process detailed below. Participants received $150 gift cards to compensate them for their time. Parents of adolescents interviewed were also offered $75 gift cards for their time coordinating transportation to and from the interviews.

Card sorts are used to understand preferences and how participants understand a particular issue (Conrad & Tucker, 2018). Card sorts were used during the interviews by asking participants to complete ranking and sorting activities with paper cards in response to certain questions. Interview questions for this study that used card sorting included: *Who are you most comfortable talking to about sex and relationships? Where do you feel safe talking about these topics? and What sexual health topics are you most interested in learning about?* The cards included possible responses to these questions, and participants were asked to move the cards to their desired order and describe their choices.

### Analogous Experiences

The analogous experience method is used to generate new insights or questions by examining seemingly unrelated experiences, interactions, and products (IDEO.org, 2015). The co-authors identified specific activities, behaviors, and feelings related to the design challenge and then identified experiences that served as positive examples and non-examples. Some of these were experiential (i.e., involved active engagement) and others were observational (i.e., did not involve active engagement). For example, museums were identified as an analogous educational experience for young people. Therefore, one of the co-authors visited a hands-on science museum and spent time observing youth interacting with exhibits while taking notes on key questions such as: *How do youth choose what to explore first? What happens when they have a question? How much time do they spend on each topic? What are they spending their time on (reading, practicing, etc.)?* Other analogous experiences included conducting observations at a juvenile justice center, observing students during school lunch hours, and participating in workshops at a global health innovation conference.

### Rapid Prototyping

The purpose of rapid prototyping is to create tangible representations of ideas to share with stakeholders and quickly gathering feedback to inform decision-making (Dow & Klemmer, 2011). These sessions consisted of showing participants the prototypes and asking questions to assess the desirability of the solution, their initial impressions, and collect initial satisfaction data. Sample questions include: *What do you like/dislike about this? What do you think this is for? Who do you think it is for? What is confusing or unclear about this?* Prototyping sessions lasted between 10 and 30 min per session. Eight rounds of prototyping were conducted, with two to four prototyping sessions conducted per round prior to analyzing data and updating the prototypes. Feedback was gathered on multiple concepts simultaneously during each session.

**Fig. 1:**
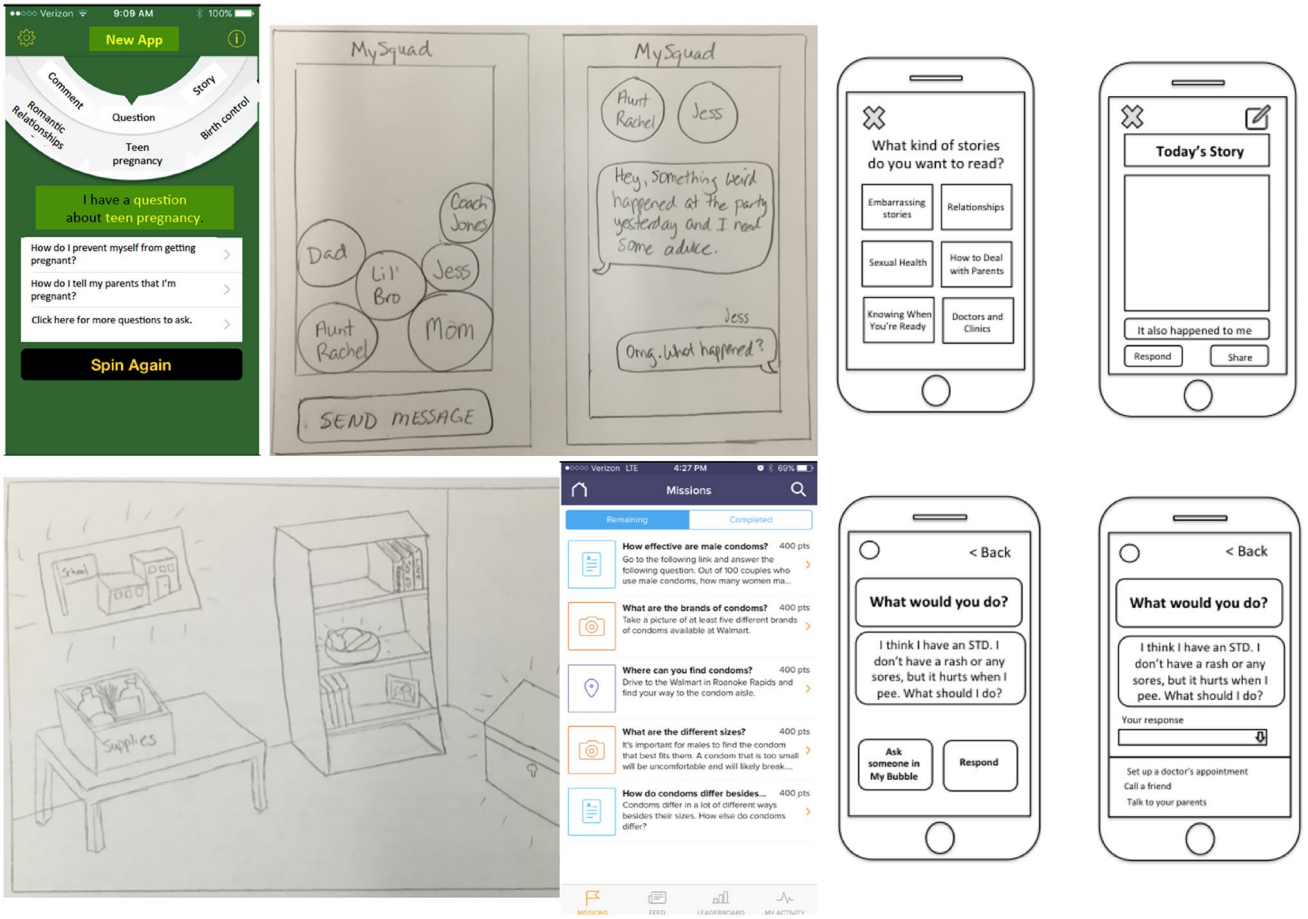
Initial prototypes. Six prototypes are shown representing different concepts, including question and answer app, a group messaging app, a story app where you can choose what topics to read about, an exploratory digital game, a virtual scavenger hunt, and an app presenting scenarios and options for responding to each scenario

Fifteen distinct concepts were tested during initial rapid prototyping sessions. These concepts were then narrowed down and refined based on feedback from these sessions. Some of the prototypes created for this method were hand-drawn, and some of them were created using basic software programs (e.g., Microsoft Word, Microsoft PowerPoint) and printed. Examples of initial prototypes are shown in Fig. 1. These example concepts include a sexual health question and answer app, a messaging app to contact trusted individuals with sexual health questions, a sexual health themed scavenger hunt, and an interactive sexual health game. In later stages, higher fidelity prototypes were developed to understand more complex behavior and concepts, such as clickable prototypes to test potential app functionality.

### Co-creation

**Fig. 2:**
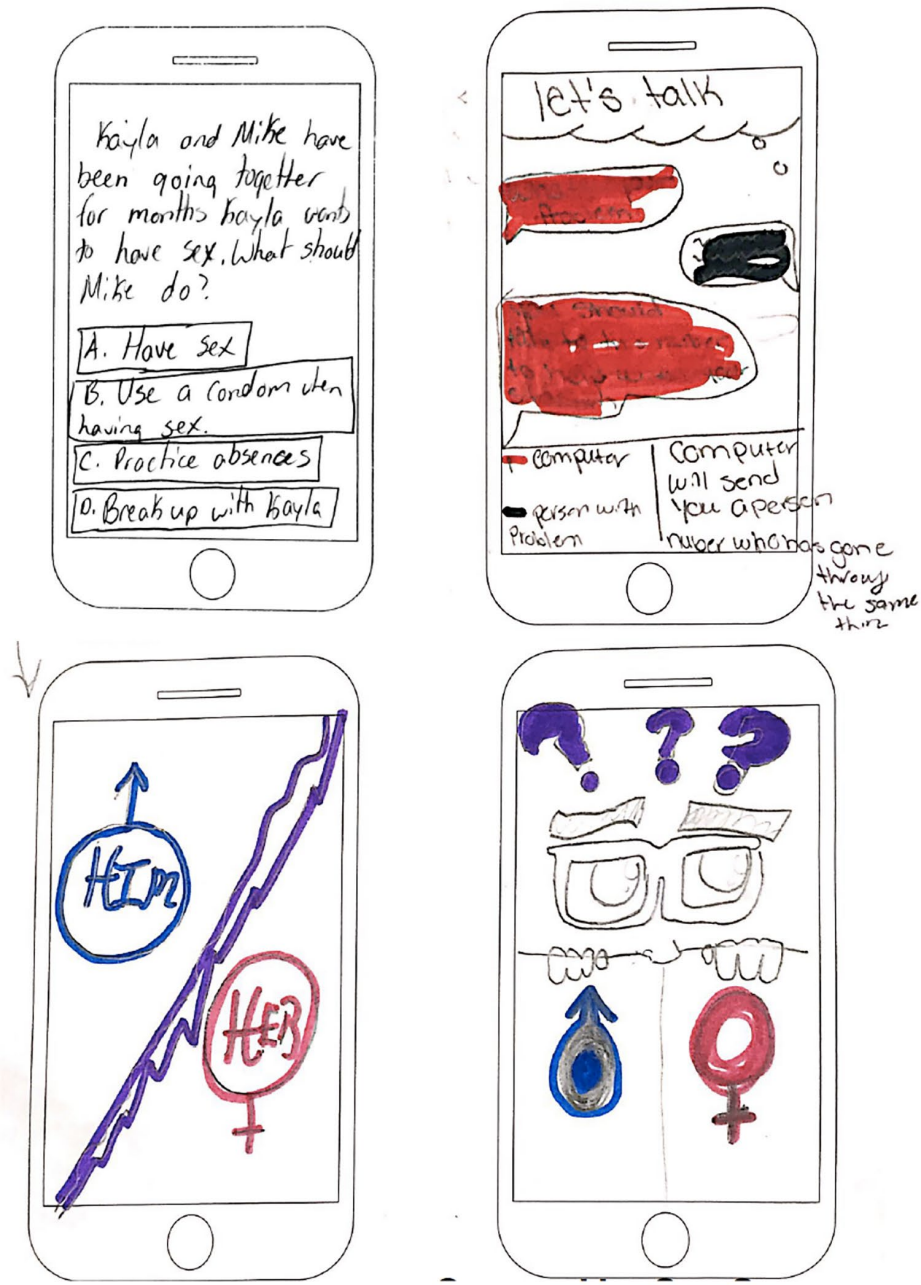
Sample designs from co-creation sessions. Four mobile app screens are shown, representing designs from two different adolescent participants. The top two screens are from one participant, and the bottom two screens are from another participant. Each screen shows hand-drawn elements including a written scenario and potential response options, speech bubbles representing dialogue between two people, and sections with content for people of different genders

Co-creation sessions are opportunities for stakeholders to be directly involved in the design of potential solutions for the topic (IDEO.org, 2015). Two co-creation sessions were held with two sets of participants new to the study. These sessions were hosted in public spaces (e.g., schools or libraries) with eight to 10 participants at each session. Participants spent 45–60 min designing their original, ideal solutions and sharing them with other participants. They were instructed to spend time (~ 10 min) individually designing and naming their solution using markers and blank cell phone screens printed on paper. Then, they presented their ideas to the group and shared feedback on others’ ideas (~ 30–50 min). Drawings were collected at the end of each session (example concepts in Fig. 2). Participants received $150 gift cards for their time.

### Data Analysis

**Fig. 3:**
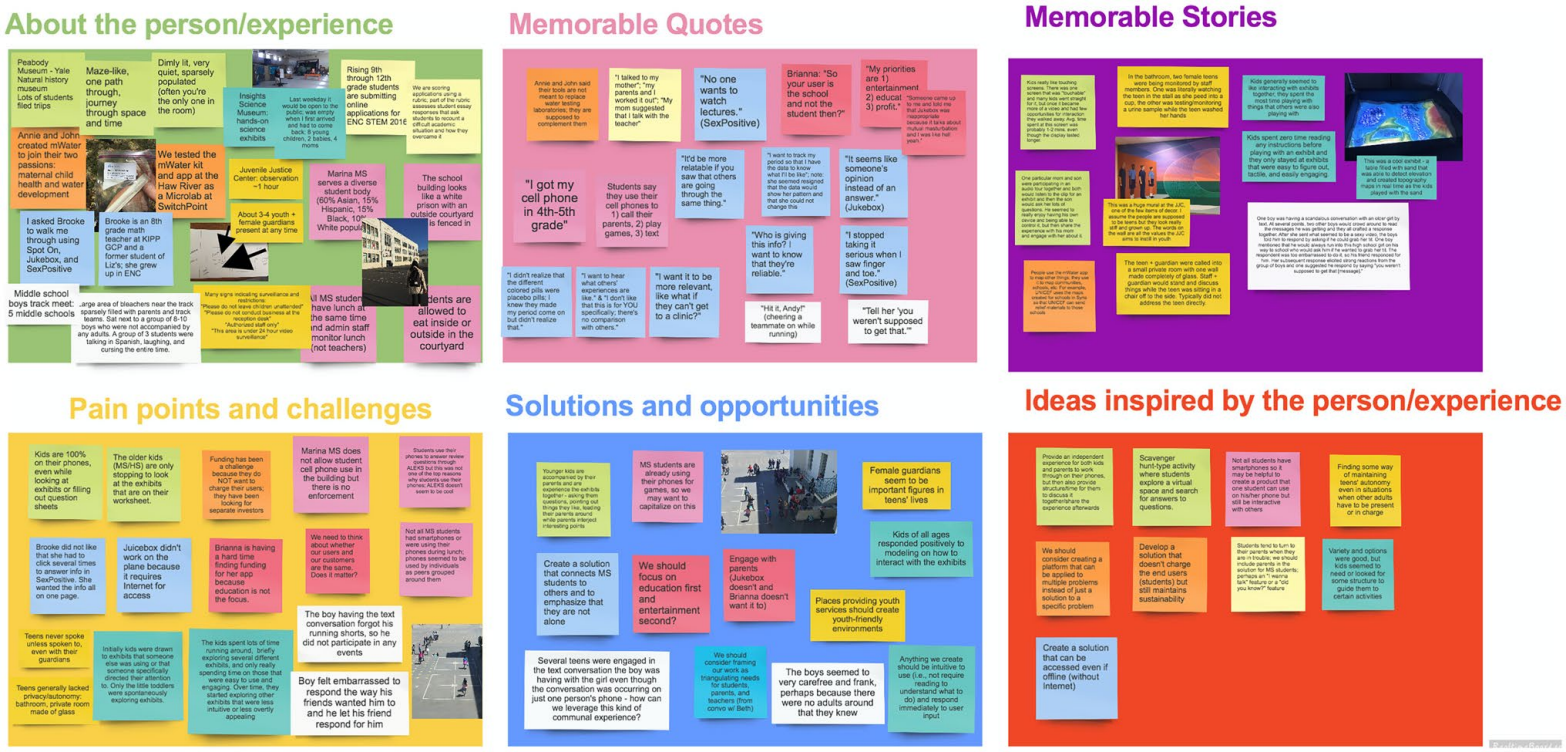
Virtual whiteboard template used to organize and analyze field notes. The template includes six boxes, each representing a different category: about the person/experience, memorable quotes, memorable stories, pain points and challenges, solutions and opportunities, and ideas inspired by the person/experience

Field notes taken by the co-authors for each activity were used for data analysis. This approach was selected based on recommendations from HCD experts and the sensitive nature of the topic. This is a suitable method for research with simple research questions (Hill et al., 2022). Field notes focused on describing the person or activity, context, memorable quotes and stories, challenges, opportunities or solutions, and any ideas inspired by the person or activity. Each team member individually transcribed these notes to sticky notes on a virtual whiteboard using a common template (Fig. 3) upon completing each activity. Each color sticky note represented a different person or experience.

Data were analyzed iteratively by the co-authors during weekly planning meetings. Co-authors CL and EC are public health professionals with formal training in qualitative research, community based participatory research, and program planning and evaluation. Co-author EC also has a background in K-12 education. Co-author VJ is an engineer with formal training in business and a background in K-12 education. All received in-depth training in HCD methods prior to completing this analysis. To analyze the data, each co-author first individually shared summaries of the data collected from the previous week to provide context for the field notes shared in the virtual whiteboard. Second, co-authors engaged in affinity diagramming (a method for grouping items into categories to identify patterns and relationships (Lucero, 2015)) and applied inductive thematic analysis (Neuendorf, 2019) by reorganizing, clustering, and labeling the clusters of sticky notes based on patterns that emerged during the discussion. Themes were identified when multiple sources of data converged around a similar idea or concept via data triangulation. The triangulated themes informed the focus for activities the following week.

Brainstorming was used after each round of data analysis to identify new areas of inquiry and concepts to explore further through prototyping (IDEO.org, 2015). Brainstorming began by focusing on a generative question, allowing time for individual brainstorming, then sharing and building on ideas together. Some sessions were conducted in-person, and some were conducted virtually using Miro, a digital whiteboard software platform. Based on the results of various data collection activities, the focus was on brainstorming solutions that could be delivered outside of school settings (preferably via a mobile app), included peer learning components, balanced structure with free exploration of content, and addressed the key emotions impacting sexual health wants and needs. New concepts generated via brainstorming were prioritized based on feasibility and impact. Those ranking highest for both were selected for rapid prototyping.

## Results

Findings from multiple HCD activities revealed important information about youth behavior, preferences, and interpersonal relationships that impacted their sexual health wants and needs, and which shaped the final prototype tested in this study. Moreover, although this study was conducted with diverse samples from three distinct geographic locations (Connecticut, North Carolina, and Texas), the core wants and needs that emerged during the analysis were similar across all groups indicating data convergence. Each theme is described in greater detail below.

### Privacy, Safety, and Credibility Are Essential

Adolescent interview participants expressed a strong need for maintaining privacy in their personal lives. They often referenced the role of social media in creating drama among friend groups and how they carefully interacted online as a result. Adolescents were diligent about seeking out private spaces when discussing sensitive issues to speak more freely with friends and trusted adults. Notably, schools and classroom settings were not considered private. During both interviews and rapid prototyping, adolescents discussed the various ways in which technology could compromise their privacy if peers or adults saw certain types of social media activity, browser history, or checked their phone messages. This deterred them from accessing certain types of content online, including sexual health resources. These adolescents also acknowledged the importance of finding credible answers to their sexual health questions and that some information online is not accurate or trustworthy. One rapid prototyping participant said: “Who is giving this info? I want to know that they’re reliable.”

### Youth Want to Learn from the Experiences of Others

Results from the interviews, analogous experience observations, and rapid prototyping activities demonstrated that youth naturally turn to their peers for information and guidance. Adolescent interview participants reported turning to their friends when they had questions about sex and relationships, even though they knew their friends may not have the right answer. However, they also reported turning to other, and sometimes older, individuals who may have been through similar experiences and could advise them on what to do. One participant said: “It’s way easier for me to talk to my cousins because they’re closer to my age and because they already talk about [sex and relationships].” Data from the analogous experience observations also showed that youth relied on information from peers when faced with ambiguous or challenging situations. During rapid prototyping, adolescents described wanting reassurance that they were not alone in their experiences and to hear how others handled a particular situation regarding sex and relationships.

### Fear of Judgment Drives Help Seeking Behavior

During interviews and rapid prototyping, many adolescents expressed a desire to turn to their parents/guardians or other trusted adults when it came to sexual health topics. However, even those with close parental relationships reported that conversations about sex and relationships were awkward, and they greatly feared disappointing their parents or being judged for their questions. This was also related to privacy concerns (described above), and the importance of having control over what information about themselves was shared publicly.

### Schools Are Not the Preferred Place to Learn About Sexual Health Topics

Adolescent interview and rapid prototyping participants reported learning about sexual health topics through various sources, including parents, peers, school health classes, and internet pornography. However, schools were reported as uncomfortable and sometimes hostile environments for seeking out sexual health information. Adolescents described how questions or conversations about sexual health topics outside of health classes could be interpreted as harassment or inappropriate behavior by school personnel, resulting in disciplinary action for the student. One adolescent said, “What we talk about in health is supposed to stay in health.” This ultimately discouraged youth from seeking out sexual health information and resources at school.

### Adolescents Prefer Mobile Apps to Websites

Adolescent interview and rapid prototyping participants reported using technology throughout the day to meet different needs (social, informational, and entertainment), and they overwhelmingly reported using mobile apps rather than websites. Websites were considered less desirable than mobile apps for certain topics due to the potential that someone may see what they were accessing online. In contrast, mobile apps were perceived to provide a more private experience. Some adolescents, especially those living in rural communities, shared that they already spent most of their time outside of school on their phones.

### Youth Preferred Authentic Story–based Content Reflecting Diverse Identities

Different types of story content were tested using rapid prototyping, starting with the use of fictional scenarios written by experts about select sexual health topics. Rapid prototyping participants immediately expressed dislike for the content and language, indicating the content was inaccurate (i.e., this is not how a teen would act) and it sounded like an adult wrote it (i.e., this is not something a teen would say). Stories were revised with adolescent input, and the revised stories received much more positive feedback. It became clear that any stories shared in the app would need to be from adolescents themselves. Rapid prototyping participants also shared it was important to provide stories reflecting a diversity of identities and experiences, including those who identify as lesbian, gay, bisexual, transgender, queer, or some other sexual orientation (LGBTQ +) youth. This was part of a larger theme to ensure the content and visual design of the app was inclusive of all gender identities.

### Youth Were Most Engaged with Content Presented in a Text-message Style Format

Rapid prototyping activities highlighted that youth typically multi-tasked while engaging with information online, including when they were watching videos and engaging with social media content. Adolescents expressed liking online videos, but they quickly lost interest in them and rarely watched videos in their entirety. Similarly, rapid prototyping participants did not want to read paragraphs of text and preferred a text message style format because it felt more inviting, personal, and looked like other messaging apps. This format also resulted in the most focused attention to the content during prototyping sessions compared to other formats.

### Youth Wanted to Safely Engage with the Content

Rapid prototyping participants reported wanting to provide support for the story writer and indicate if they had experienced something similar. Some suggestions for this included adding a “this happened to me” button or sharing their own story. The use of a commenting feature was explored, but adolescents immediately expressed concern and asked how online behaviors would be moderated to avoid bullying. It became clear that balancing the use of peer connection and support with user safety on the app would be needed to effectively serve youth.

### Final Prototype: The Real Talk Mobile App

**Fig. 4:**
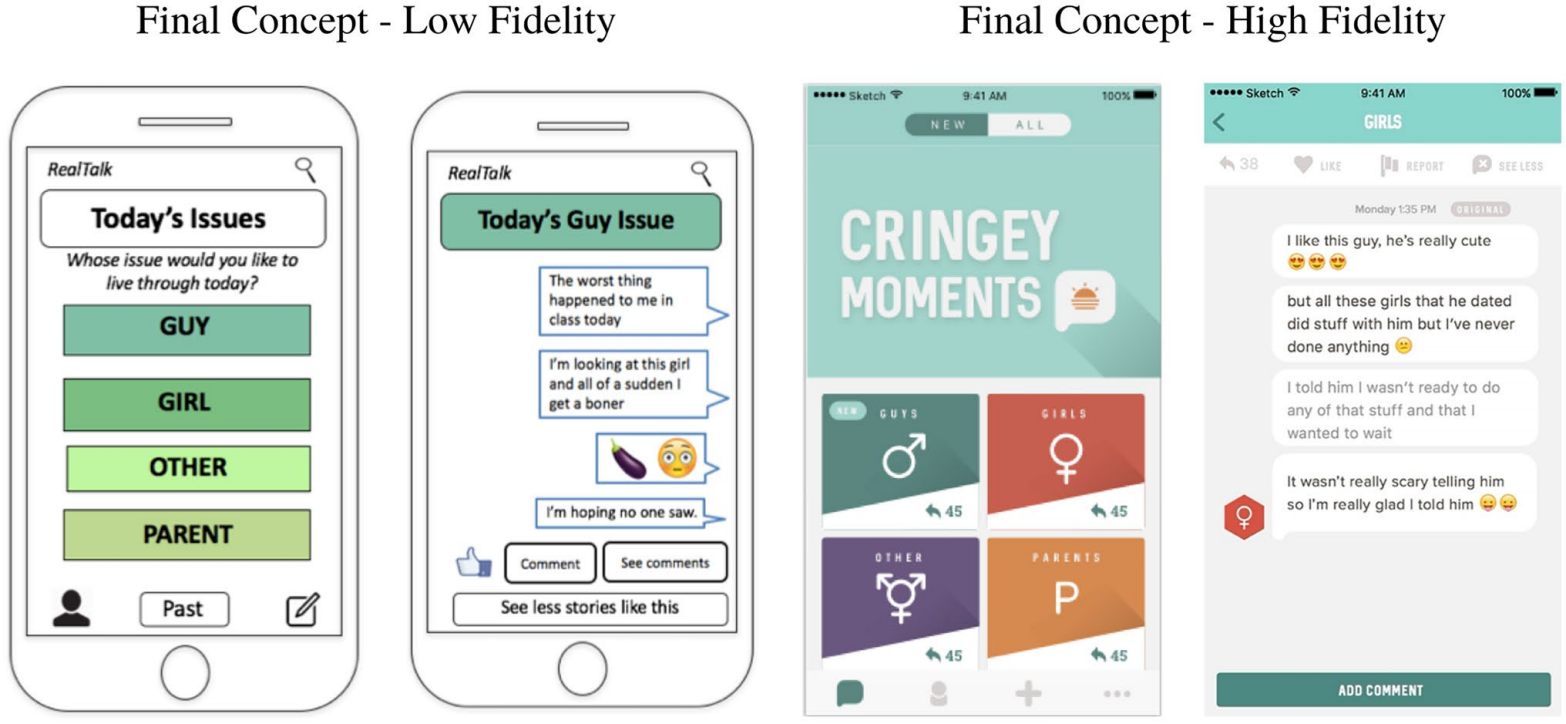
Final mobile app concepts, including a lower fidelity version showing the home screen and story screen made with Microsoft PowerPoint and a higher fidelity version showing the home screen and story screen made with professional design software

The final prototype created as a part of this study was *Real Talk*, a mobile app using authentic youth stories to deliver sexual health information and resources to adolescents 13–15 years old. Figure 4 shows an earlier, low fidelity (i.e., low approximation) version of the app interface as well as a later, high fidelity (i.e., high approximation) version of the app interface.

## Discussion

This study used a HCD approach to identify adolescents’ unmet sexual health wants and needs and translate these insights into a novel digital intervention. Findings highlighted several important insights, including the role of school settings; concerns about privacy, safety, and credibility when it came to accessing sexual health content; key emotions impacting adolescents’ access to and use of sexual health resources; and opportunities for technology to foster greater access to and engagement with sexual health content. Together, these findings led to the development of an innovative, mobile health (mHealth) intervention, the *Real Talk* mobile app.

The themes identified in this study align with the findings reported in other adolescent mHealth research in the field of SRH. For example, while conducting a feasibility and acceptability study for an STI/HIV and drug abuse prevention mHealth intervention, Cordova et al. (2018) learned that privacy was important, especially in school settings where it was difficult to take content seriously when classmates were giggling. Yet, the use of HCD methods in this study illuminated this need early on and the intervention was designed with it in mind. This represents a key benefit over traditional intervention development approaches where important requirements are not discovered until after a full intervention is created and tested. Findings from this study also represent cross-cutting themes that may be useful for research and practice involving additional health behaviors (e.g., drinking, smoking, vaping). Indeed, adolescent researchers focused on substance use (Adams et al., 2021) as well as alcohol use and suicidal behavior (O’Brien et al., 2019) reported similar findings regarding the desire for a mobile application to provide health information and social support among adolescents. Finally, findings regarding peer learning are echoed in previous research. Other SRH literature shows adolescents have responded favorably to peer-to-peer sexual health education interventions (Layzer et al., 2017; Mellanby et al., 2001). Advantages of peer education SRH interventions include adolescents perceive that information from peers is credible and trustworthy (vs. information from adults may be received with mistrust or be seen as preaching) and peer-led interventions may lead to improved outcomes for the peer educators themselves (Sriranganathan et al., 2012).

This study also makes notable contributions to the adolescent SRH field. While robust empirical and theoretical evidence exists to guide adolescent sexual health intervention development, the HCD methods used in this study demonstrate quick and cost-effective techniques for identifying the most salient factors to target for a particular population of interest. This approach allowed for a holistic understanding of context, setting, channel, content, language, emotions, and interactions that influence uptake and use of a technology-based sexual health intervention. For example, while health education interventions are commonly delivered in schools, this study describes important limitations of delivering sexual health content in this setting. Similarly, insights regarding how content should be delivered (e.g., providing stories rather than facts, using authentic stories rather than fictional scenarios written by adults, and using a text-message style format) were a direct result of engaging in HCD activities with youth and modifying the intervention according to their feedback.

### Future Directions for HCD in Public Health

This study also makes an important contribution to the broader intervention development field. Information about the HCD process followed is openly accessible online; thus, others can apply these same methods to develop and adapt future interventions, as well as to better understand populations of interest. There have been several recent studies using the HCD process to design interventions, adapt interventions, and understand people and contexts among adult populations (e.g., Ector et al., 2020; Suen et al., 2021; Van Der Westhuizen et al., 2020). Importantly, HCD provides useful tools for developing interventions but is not meant to replace other common methodological approaches; HCD can be combined with other approaches to generate ideas and design solutions aligned with the people’s wants and needs.

This work demonstrates the benefits of using HCD for intervention development to advance the field of adolescent SRH. Additional strengths of this project include (1) detailed documentation of the process so that others can apply HCD methods in their future research, (2) the unique recruitment strategy based on identifying extremes and mainstreams for interviews, (3) the intentional rapid prototyping strategy where multiple ideas were tested with adolescents quickly before selecting one idea to develop further, and (4) deliberate involvement of adolescents in co-creating the intervention; the data they provided informed the content, format, and user experience of the intervention developed to explicitly address these wants and needs. Researchers and practitioners are encouraged to incorporate HCD methods into their work.

### Limitations

One key limitation involved constraints around funding and resources. Technology-based interventions generally require greater investment of resources during initial development compared to other modes of delivery, with the expectation that technology platforms become significantly more cost-effective to manage over time as the program scales to serve more participants. The visual design and quality of user experience for a digital platform also play a critical role in enhancing or inhibiting the adoption and use of digital tools (Lazard & Mackert, 2015). Given limited resources, it was not possible to prototype or build all the features adolescents desired while still providing a high-quality mobile app experience. This is one of several existing barriers to effectively scaling interventions that warrants continued attention in the field (Fagan et al., 2019). The funding for this project was also earmarked specifically for SRH, constraining the app’s focus even though many adolescents wanted additional resources for topics such as mental health and bullying.

A second limitation involved real world constraints related to working with a younger adolescent population. The original intent was to work with middle school age children, including those who may be as young as 11 or 12 years of age. However, gathering personal information from users to properly assess reach and engagement proved to be a challenge based on The Children’s Online Privacy Protection Act (COPPA), which requires parental consent be obtained prior to collecting certain types of information online from children under the age of 13 (Federal Trade Commission, 1998). For example, many participants wanted individual accounts for a more personalized app experience, but COPPA prevents the collection of personal information required to generate log-ins (e.g., names, email addresses, zip codes) for those under 13 without parental consent. The added step of obtaining parental consent prior to accessing the app interrupted an otherwise quick and seamless onboarding experience. This also further excluded youth who may have wanted to use the app but did not want their parents knowing that they were downloading an app with sexual health content. Several options were considered for handling personal data, and ultimately, it was determined that it would not be feasible to create a technological solution that met both adolescents’ wants and the ethical standards set for this project. Therefore, the project pivoted to serving adolescents ages 13 to 15.

## Implications for the Future

This study provides an in-depth example on how to apply HCD methods to develop novel adolescent sexual health interventions. Strategies described here can be applied at various stages of the intervention development lifecycle to generate data-driven, actionable insights. Using these approaches, researchers and practitioners can strengthen their own intervention development efforts to improve the reach, adoption, and implementation of sexual health interventions.

### Supplementary Information

Below is the link to the electronic supplementary material.

Supplementary file1 (DOCX 10 KB)

## Data Availability

The data used for this study are available from the corresponding author, CL, upon reasonable request.
