# Supplementary material for: Using Human-Centered Design Strategies to Identify Unmet Adolescent Sexual Health Wants and Needs
Source: Prev Sci. 2023 Jun 23;24(Suppl 2):174–84. doi: 10.1007/s11121-023-01559-5 (PMC10764404; doi:10.1007/s11121-023-01559-5)
Supplement: Supplementary file 1 — Supplementary file1 (DOCX 10 KB) [file 11121_2023_1559_MOESM1_ESM.docx]

**Appendix A - Interview Guide**

1. Tell us about yourself. (Did you grow up around here? Where do you live now?)
   1. **For adults:** How long have you been in your current position? What brought you to this position? What’s been the most surprising/challenging part of your job?
2. How would you describe your childhood home? What was it like to grow up there?
   1. **For youth:** Tell us about how you use technology on a daily basis.
      1. What sorts of things do you use technology for?
      2. What apps do you like to use most?
      3. Can you show me your phone and some of your favorite apps on it?
      4. When do you use your phones with other peers?
3. Tell us about the first time an adult tried to explain sex to you.
   1. **For youth:** How was this experience?
4. How did you really learn about sex and pregnancy?
   1. **For adults:** Tell us about conversations about sex you have with your kids or students.
      1. What parts are easy to explain?
      2. What parts are hard to explain?
      3. What do you wish you could say?
   2. **For youth:** How should parents talk to their kids about sex and pregnancy?
      1. How should teachers do it?
      2. Who or what is actually the best person or thing to learn from?
5. What is the worst, most uncomfortable way to learn about sex and pregnancy?
6. Here is an app [Juicebox, SexPositive] for you to try. Talk me through your experience as you use it.
7. What is one thing you wish someone or something had told you about sex or pregnancy as a 13-year-old?
8. Card sort: sort which adults you speak to for different scenarios
